# Promoting Well-being Among Informal Caregivers of People With HIV/AIDS in Rural Malawi: Community-Based Participatory Research Approach

**DOI:** 10.2196/45440

**Published:** 2023-05-11

**Authors:** Laura Sbaffi, Efpraxia Zamani, Khumbo Kalua

**Affiliations:** 1 Information School University of Sheffield Sheffield United Kingdom; 2 Blantyre Institute for Community Outreach Blantyre Malawi

**Keywords:** informal caregivers, HIV/AIDS, rural Malawi, health advisory messages, mobile phone

## Abstract

**Background:**

People living with HIV/AIDS and their informal caregivers (usually family members) in Malawi do not have adequate access to patient-centered care, particularly in remote rural areas of the country because of the high burden of HIV/AIDS, coupled with a fragmented and patchy health care system. Chronic conditions require self-care strategies, which are now promoted in both developed and developing contexts but are still only emerging in sub-Saharan African countries.

**Objective:**

This study aims to explore the effects of the implementation of a short-term intervention aimed at supporting informal caregivers of people living with HIV/AIDS in Malawi in their caring role and improving their well-being. The intervention includes the dissemination of 6 health advisory messages on topics related to the management of HIV/AIDS over a period of 6 months, via the WhatsApp audio function to 94 caregivers attending peer support groups in the rural area of Namwera.

**Methods:**

We adopted a community-based participatory research approach, whereby the health advisory messages were designed and formulated in collaboration with informal caregivers, local medical physicians, social care workers, and community chiefs and informed by prior discussions with informal caregivers. Feedback on the quality, relevance, and applicability of the messages was gathered via individual interviews with the caregivers.

**Results:**

The results showed that the messages were widely disseminated beyond the support groups via word of mouth and highlighted a very high level of adoption of the advice contained in the messages by caregivers, who reported immediate (short-term) and long-term self-assessed benefits for themselves, their families, and their local communities.

**Conclusions:**

This study offers a novel perspective on how to combine community-based participatory research with a cost-effective, health-oriented informational intervention that can be implemented to support effective HIV/AIDS self-care and facilitate informal caregivers’ role.

## Introduction

### Background

Malawi is a landlocked, low-income country located in the southeast of Africa, with a population of approximately 19 million people [1]. The population’s life expectancy, although in steady increase, is set at 65.6 years, in net discrepancy with, for example, at 78.5 years in the United States or at 81.4 years in the United Kingdom [2]. Malawi exhibits the 9th highest HIV/AIDS prevalence rate in the world among people aged 15 to 49 years [3,4]. Women have a higher prevalence rate (10.4%) than men [4], reflecting a gender disparity in the risk of contracting HIV/AIDS [5,6]. The HIV/AIDS epidemic is also heterogeneous in the country, with the prevalence in the southern region (16%) being more than double than that in the north (7.4%) [7]. Although the incidence of HIV/AIDS-related deaths in Malawi has decreased by 55% since 2010, this condition remains the main cause of mortality in the country [8].

HIV/AIDS is a chronic, communicable disease that requires considerable self-care [9] and is associated with a range of comorbidities, including hypertension, stroke, cancer, and diabetes [10,11] among others. In this context, self-care refers to the daily activities that patients engage in to monitor HIV/AIDS-related symptoms, adhere to treatment, and follow healthy habits [12]. Extensive support is required to help patients make informed decisions, adjust their behavior, and adapt to their condition [13]. Angwenyi et al [14] emphasized that the needs of people living with HIV/AIDS changed with the introduction of antiretroviral (ARV) therapy as they moved away from basic nursing care toward more complex psychosocial, livelihood, and nutritional support. However, the Malawi national health care system is still underdeveloped and understaffed, with satisfactory health and social care available only in urban areas [15]. In addition, Malawi, similar to other low-income countries, has fragmented transport and communication means connecting rural and urban areas, which makes the delivery of health care services to remote regions particularly difficult [16]. However, 83% of the country’s population lives in rural areas [17], where the paucity of health facilities has not only negative implications on diagnosis and continuity of care but is also compounded by socioeconomic differences, poverty, and low literacy levels that result in social exclusion [18,19]. The World Health Organization [20] health statistics overview stated that countries with fewer than 10 physicians and 40 nurses or midwives for every 10,000 people are considered to face a serious shortage of health care professionals. This places Malawi, where each physician is expected to serve >50,000 individuals [21], solidly in the group of countries considered to have a serious deficiency of health care professionals and hence, whose population has less access to essential health services [20]. This shortage of health care resources has also been identified as a major hindrance to the large-scale implementation of ARV therapy in the country [22]. HIV/AIDS self-care is a relatively new concept in sub-Saharan African countries, and many African health care systems are undergoing a phase of transition from disease-specific to more integrated health services [14]. In recent years, Malawi introduced several clinics for HIV/AIDS tracing [23] and integrated care with treatments for noncommunicable diseases, such as asthma, epilepsy, and mental illnesses; however, they are only limited to a few districts and more densely populated areas [15].

Against this backdrop, the burden of looking after a person affected by HIV/AIDS in a rural area falls primarily on their families and the local community. However, caring for a patient with HIV/AIDS requires effort and time, as well as basic knowledge of care, which can be acquired through access to quality information on symptom monitoring, how to administer medications, handle adverse effects experienced by patients, adopt and maintain a healthy diet, and more [24,25]. Most caregivers are not trained to undertake this role, and when appropriate information and advice in relation to HIV/AIDS and its management are missing or inaccessible because of the lack of a functioning health care system, care may involuntarily cause additional harm to the patient and increase the burden on caregivers [26].

In light of this, we started working with informal caregivers in the Mangochi district within the context of an initial scoping study in June 2019. A 2-day workshop with informal caregivers of people with HIV/AIDS, social workers, rural community chiefs, academics, and local nongovernmental organizations gave us the opportunity to discuss caregivers’ needs, observe and understand the challenges they face, as well as identify potential solutions to support them in their everyday life and to improve their well-being and quality of life. This scoping exercise, fully discussed by Zamani and Sbaffi [27], indicated that informal caregivers lacked access to timely and basic information on how to look effectively after their loved ones affected by HIV/AIDS. They shared with us that they would consider it beneficial to receive regular and reliable information on key health aspects related to HIV/AIDS management, which would partially subsidize the lack of a local, functioning health care system. Caregivers confided that they would welcome advice and guidance on daily care practices, which would help them manage their lives and those of their loved ones in nonemergency situations. We also discussed with them a viable and effective solution for conveying such information in line with local communities’ dynamics and available communication infrastructures. The widespread habit of sharing a mobile phone and airtime among members of rural communities led us to the decision, in agreement with the caregivers, of using the WhatsApp voice-recording function to deliver prerecorded HIV/AIDS-related health advisory messages at caregiver support group meetings, which are considered a social focal point of local rural communities. In fact, in Malawi, many informal (or family) caregivers are supported by so-called community home–based care (CHBC) programs, which were first introduced in sub-Saharan African countries with the advent and diffusion of ARV therapy in the 1980s [28]. CHBC programs offer a wide range of activities and services, including identifying people (both those affected by HIV/AIDS and their caregivers) requiring CHBC to provide basic nursing care. They also actively advocate for lessening the stigmatization and discrimination of people living with HIV/AIDS [29]. The CHBC policy defines the role of caregivers within the context of the *5Rs* (readiness of services, retention of health care staff, responsibility of society and government, referral mechanisms, and reporting of results) delineated by the Government of Malawi in their Health Sector Strategic Plan [30], to promote equity and quality in health care. Among the services provided by CHBC programs is the establishment and coordination of support groups for informal caregivers, which are then led and maintained by a volunteer caregiver or local community chief. This research leveraged the local Namwera CHBC program to promote participation of caregivers in the study.

The decision to use WhatsApp as the means to deliver the health advisory messages stemmed from two main considerations as follows: (1) this social media platform is the most widely used among people living in rural Malawi (social workers and caregivers—personal communication) and (2) research shows that WhatsApp is used by health care professionals in sub-Saharan African countries to share information with each other and with patients [31], and health-related information received via this platform can positively influence behaviors regarding family health care [32].

### Overview of Community-Based Participatory Research Designs in Health

Participatory design is a form of research that establishes frameworks of participation encouraging individual voice and representation rather than trying to rearrange the sociopolitical environment in which the research is based [33]. Community-based participatory research (CBPR) is a method of inquiry that involves community members as equal partners (ie, not just as participants or recipients) in all phases of the research process, with the goal of educating, improving practice, and promoting social change and positive health outcomes [34,35]. In researching communities that experience marginalization, this approach is considered particularly valuable because it encourages the establishment of mutually respectful relationships with such communities and the sharing of control over health and social care outcomes [35]. CBPR has a particularly important role in studies focusing on public health and social determinants of health [36,37] and, over the last 20 years, has gained substantial traction in low-income countries with a particular focus on health equity [38], also in an attempt to respond to the United Nations Sustainable Development Goal 3 “Ensure healthy lives and promote well-being for all at all ages” [39]. However, the “western” understanding of the processes and factors linking community-based collaborative initiatives with changes in measurable determinants of health outcomes is still inadequate and requires effective and web-based processes of knowledge exchange between researchers and knowledge users, where “knowledge uses” are defined as “individuals directly affected by research and inclusive of those who occupy a range of positions in health systems: funders, health system and policy decision-makers, health care providers, patients, and family members” [40]. Sustainable health-related interventions adopting CBPR require context-sensitive approaches, openness, and actions that should be determined by those who ultimately benefit from such interventions [41]. Effective CBPR projects have also demonstrated to increase a sense of community ownership and trust in the research, which leads to more successful recruitment and retention of participants [42,43] and to the participants’ willingness to “champion” the research beyond its original scope [44].

There is unequivocally an extensive body of research focusing on the benefits of CBPR, but much less has been produced on the challenges associated with this approach, particularly in relation to ethical considerations [45]. Kwan and Walsh [46] highlighted, among others, difficulties in balancing community values, needs, and identity with those of the researchers; negotiating dynamics and relationships; working with stigmatized and marginalized populations; and facilitating sustainable action emerging from the findings. None of these aspects are easily achievable, and a rigorous planning process including discussions and shared decision-making is therefore essential before engaging in community-based research [47].

In the context of conducting CBPR in an African setting, as in this study, attention must also be paid to the deeply rooted way or philosophy of life that influences societies and individuals alike. This philosophy is known as *uMunthu* and can be distilled into a sense of solidarity and kinship with one another and living in harmony within the communities [48]. uMunthu has been leveraged in a variety of Africa-based CBPR projects to facilitate the accomplishment of the desired research outcomes. To fully engage with this philosophy, and formulate a viable CBPR project, researchers from outside the communities must identify themselves “as part of a wider community that encompasses designers from inside and outside who together derive a communal existence” [49]. In CBPR projects, a community comes together when work is to be done to jointly accomplish shared goals and values. This happens naturally in uMunthu, as part of the spirit of unity integral to the community [50]. Consequently, for health interventions to have a real impact in African contexts, they need to address not only the individual or specific group of interest but also the community as a whole [51].

CBPR was selected as the research approach in this study to engage informal caregivers of people with HIV/AIDS in a process in which they had something to offer, that facilitated mutual learning between the researchers and the communities, that was aligned with local interests, and that had the potential to lead to social change.

### Research Aim and Objectives

On the basis of our initial scoping exercise conducted in June 2019 [27] and through the application of a CBPR approach, the overarching aim of the study was to support informal caregivers of patients with HIV/AIDS in their role and everyday life via the delivery of prerecorded, HIV/AIDS-related health advisory messages, and specifically to achieve the following research objectives (ROs):

1. RO1: assess caregivers’ perceptions of the usefulness and applicability of the health advisory messages to their daily life and caring responsibilities.
2. RO2: evaluate the self-assessed, everyday impact of the messages on informal caregivers’ and their families’ well-being.
3. RO3: consider the potential for a long-term impact of the messages on informal caregivers and the wider community.

In the following sections, we first present the method of the study, offering details on the study context, research design, approach to data collection and analysis, and ethical considerations. We then provide an overview of our findings, discuss them in relation to the existing literature, and summarize the study’s contribution. We conclude with a description of our study’s limitations and avenues for future research.

## Methods

### Study Context

The Mangochi district, where this study was conducted, has a population of approximately 1 million people mostly located in rural settlements and is the third hardest hit by HIV/AIDS in Malawi after Lilongwe and Blantyre. Tourism, high illiteracy levels, and deeply rooted cultural practices are believed to have contributed to the high levels of HIV/AIDS in the district [4,52]. In addition, a poor transportation system and lack of medical staff are other reasons why remote and rural communities are unable to access accurate medical advice [53,54]. In this study, we focused specifically on Namwera, a large rural area located in the southeastern part of Mangochi, bordering Mozambique. The study was conducted by a joint team of researchers from the University of Sheffield in the United Kingdom and the Blantyre Institute for Community Outreach in Malawi, with the support of the Namwera AIDS Coordinating Committee (NACC), a local organization established in 1996 to sensitize the community to the risks of HIV/AIDS and sexually transmitted diseases. The Namwera NACC also operates a CHBC program for the development of HIV/AIDS informal caregivers support groups in the area. Such support groups meet monthly, and the informal caregivers who attend them have the opportunity to share their lived caring experiences and exchange acquired knowledge of HIV/AIDS and caring practices. In this paper, we report on a study that we conducted with members of 5 informal caregivers support groups established by the Namwera NACC CHBC program.

### Participants

There are 16 HIV/AIDS informal caregivers support groups in Namwera that have been established, trained, and supported by NACC and its CHBC program over the last 25 years. For the purposes of this study, we worked with 5 of these support groups closely located to the NACC Namwera center for convenience purposes (to allow participants to walk to in-person activities, such as the summative workshop): Balakasi, Chingwenya, Lusangwisi, Namawerenga, and Somba. The membership of the groups and the number of participants in the study are summarized in Table 1.

**Table 1:** Participating HIV/AIDS informal caregivers’ support groups^a^.

Name of support group	Year established	Women, n (%)	Men, n (%)
Balakasi	2008	18 (100)	0 (0)
Chingwenya	2008	16 (80)	4 (20)
Lusangwisi	2003	19 (100)	0 (0)
Namawerenga	2014	10 (91)	1 (9)
Somba	2009	18 (69)	8 (31)

^a^Total participants: 81 (86%) women and 13 (14%) men.

All support group members aged ≥18 years who were undertaking caring responsibilities for a person living with HIV/AIDS at the time of the study were invited to participate by Namwera NACC representatives who approached them in person at individual group meetings. Overall, 94 informal caregivers agreed to participate in this study.

### Study Design

Our study followed the principles of CBPR, whereby the research design and our research instruments were cocreated with our research participants [55]. As discussed in the *Introduction* section, we devised, together with caregivers, an intervention addressing their need for support with daily caring activities based on the formulation and dissemination of HIV/AIDS-related health advisory messages. At the scoping 2-day workshop organized in June 2019, it was established that the messages would be delivered via the audio WhatsApp function to each of the 5 support group leaders, who would then broadcast them via Bluetooth speakers at their respective monthly meetings. The project time constraints implied that a maximum of 6 messages could be devised and delivered. We started the work for this project in August 2020 by organizing initial meetings with caregivers, social workers, area development committees, village development committees, and the leaders of the 5 participating support groups to contribute to the identification of the topics for the advisory messages, to train support group leaders on consent principles (required as part of the ethics approval process), and to instruct them on the use of WhatsApp audio function and solar batteries for mobile charging during support group meetings (this equipment was necessary owing to the lack of electricity and power infrastructure in some villages). To formulate the content of the advisory messages, we worked together with 2 local medical physicians (general practitioners), 1 social care worker, and 2 support group leaders, which led to the development of 5 messages on topics closely related to the management of HIV/AIDS. This process involved several iterations over a period of 5 months, during which support group leaders would provide feedback on the clarity of the language and tone adopted by the messages. The 6th message was conceived later to give participating caregivers the time to take stock of the previous ones and identify an additional topic that would represent an appropriate integration with the initial 5 messages. Individual support group leaders held discussions with members of their respective groups and most caregivers agreed that a valuable final message would be in relation to safe pregnancy and motherhood in the context of HIV/AIDS. The prerecorded audio messages were distributed both in Chichewa, the official native language of Malawi, and Chyao, the most common local dialect. Their duration varied between 9 and 15 minutes, and they were recorded in layperson’s language in consideration of the poor literacy levels of the rural communities.

Each message was distributed monthly to the 5 participating support groups between November 2020 and March 2021, with the last message distributed later in June 2021, as shown in Table 2.

**Table 2:** List of health advisory messages delivered monthly to caregiver support groups’ members.

ID	Distribution period	Advisory message topic
1	November 2020	COVID-19 and HIV/AIDS (Multimedia Appendix 1)
2	December 2020	HIV/AIDS and nutrition (Multimedia Appendix 2)
3	January 2021	HIV/AIDS and emotional and social support (Multimedia Appendix 3)
4	February 2021	ARV^a^ dosage, side effects, and myths (Multimedia Appendix 4)
5	March 2021	First aid and safety in caring for HIV/AIDS patients (Multimedia Appendix 5)
6	June 2021	Safe motherhood and HIV/AIDS (Multimedia Appendix 6)

^a^ARV: antiretroviral.

To achieve our research objectives, we used qualitative data collection methods that helped us capture informal caregivers’ experiences with this intervention and to explore and understand their views and opinions. Namely, we collected qualitative data via individual face-to-face interviews with 94 participating caregivers and coordinated a final summative workshop with 3 members of each support group. The individual interviews followed a semistructured format based on predefined questions that were used solely for probing purposes (Multimedia Appendix 7), which allowed us to delve into the perspectives and experiences shared with us by each participant. The individual interviews with caregivers were conducted 2 weeks after the broadcasting of each message, except for the last message, which was only distributed at the end of the study period. Before each interview, verbal informed consent was collected from the interviewees, and the purpose of the study was previously explained and discussed during the support group meetings by each group leader. The interviews were conducted either in Chichewa or Chyao, depending on the preference of the interviewee. The interviews were recorded, manually transcribed, and translated into English. The interviews included demographic questions and questions on the participants’ opinions on the relevance, clarity, and usefulness of the advisory messages. The transcripts were manually analyzed following the thematic analysis by Braun and Clarke [56]. First, we familiarized ourselves with the interview material and then generated initial codes by identifying themes within each interview. We then reviewed the evolving coding scheme by grouping some emergent themes into broader concepts. We then examined our themes against each other to ensure that each was distinct from the rest, and then compared them within and across each interview to identify similarities, differences, and possible discrepancies and refine our interpretations [56]. While doing so, we kept copious notes and memos to ensure a fit between the empirical material and our interpretations.

Following the interviews, at the end of the study period, we held a summative workshop with informal caregivers from the original sample, support group leaders, community chiefs, and general practitioners (25 people in total). The purpose of this workshop was to assess the impact of the intervention on informal caregivers, the people they care for, and their communities, with a specific focus on the perceived influence of the messages on everyday caring routines and the potential for long-term impact. The workshop was also designed to facilitate a dialogue between caregivers and health care providers about caring responsibilities and other forms of support (in addition to the intervention carried out in this study) available to rural communities affected by HIV/AIDS.

### Ethics Approval

Before engaging with informal carers, we secured ethics approval from the University of Sheffield (application number 032332), following the procedure that applies to research conducted outside the United Kingdom, considering the absence of a similar procedure in Malawi, and with the view to undertake a “belt-and-braces” approach [57] that would avoid ethical oversight. This approach was adopted in light of the sensitive nature of some of the aspects emerging from the interviews and to guarantee our compliance with local social rules and community values.

### Informed Consent

We secured caregivers’ consent (either written or verbal depending on the literacy level of the caregiver) to participate in the research and informed them that their participation in the study was entirely voluntary and that they had the right to withdraw from it at any time without providing a reason. We also encouraged them to attend the interview accompanied by a trusted person if they preferred it and could choose to interrupt the interview as often as required. Ethics clearance also entailed that none of the participants would be identified by name or by another marker that could possibly risk their identification. All participants were provided with information on the project in writing (Chichewa, Chyao, and English) and this information was offered verbally for those who were unable to read. Caregivers were also incentivized to participate via the regular distribution of food packs. Throughout the project, the nature of our interactions with caregivers was guided by the advice of social workers and community chiefs to minimize unwarranted behavior and not to overstep social boundaries.

## Results

### Demographic Information

Table 3 summarizes the demographic information of the participants. Women were by far more numerous than men, representing >86% (81/94) of the sample. More women than men attend support groups in general, and informal discussions with the group leaders revealed that men tend not to join, as they are afraid of not getting into other sexual relationships once their close contact with HIV/AIDS is disclosed. Only the Somba support group had considerably more men compared with the others because of a door-to-door initiative by 1 man who wanted to convey the importance of attending such groups for men. Two-thirds of the respondents (50/81, 62% women and 12/13, 92% men) were either married or in a long-term relationship. Although 51% (48/94) of the respondents reported having had some form of education, when asked a direct question about their ability to write and read, 62% (58/94) responded they had none. All men were farmers, as compared with 78% (62/81) of the women who also worked in independent businesses or as casual workers in addition to being farmers themselves. Of those who disclosed their age, 86% (67/78) were aged ≤54 years and 71% (67/94) had good or very good health (self-assessed measure). About two-thirds (64/94, 68%) of the participants were affected by HIV/AIDS, but they functioned as caregivers of members of the family in worse health conditions than their own. This self-assessed measure of health status was provided as a comparison with the health of the loved ones they were looking after.

**Table 3:** Demographic characteristics of the study participants (N=94).

Characteristics			Values, n (%)
**Gender**			
	Men	13 (14)	
	Women	81 (86)	
**Marital status**			
	Married or with partner	62 (66)	
	Widowed	10 (11)	
	Divorced	13 (14)	
	Single	9 (10)	
**Education**			
	Not educated	46 (49)	
	Primary school level	42 (45)	
	Secondary school level	6 (6)	
**Occupation**			
	Farming	76 (81)	
	Casual worker	9 (10)	
	Small business	6 (6)	
	No occupation	3 (3)	
**Perceived health**			
	Very good	25 (27)	
	Good	42 (45)	
	Poor	20 (21)	
	Prefer not to say	7 (7)	
**Age group (years)**			
	18-24	4 (4)	
	25-34	11 (12)	
	35-44	30 (32)	
	45-54	22 (23)	
	55-64	6 (6)	
	≥65	5 (5)	
	Prefer not to say	16 (17)	

More than half of the participants (52/94, 55%) were looking after a child or multiple children with HIV/AIDS, approximately 18% (17/94) looked after their partner, and the remaining cared for other relatives in their family (eg, sibling, parent, or grandparent). The amount of time they spent looking after a person living with HIV/AIDS spanned from a few months to 20 years, with 52% (49/94) having performed their caregiving role for a year or less. Only 6 participants had access to a smartphone, as the majority shared either a mobile phone or airtime with other members of their community.

### Analysis of Qualitative Data From Interviews

The interviews lasted on average 35 minutes (SD 12 min; ranging from 20 min to 1 h and 10 min) and focused on the interviewee’s impressions of the health advisory messages. Although the interview script included many open-ended questions, the responses from the participants tended to be short and factual, without exceeding the elaboration of the statements made, which is considered a limitation of this study. The thematic analysis of such answers was performed by LS, but the other coauthors independently cross-checked 40% each of the interviews content to verify the themes identified. Any disagreements were discussed and resolved by the research team. In this study, the themes related to (1) the daily application of message advice, (2) long-term perceived benefits of messages, and (3) quality and clarity of messages were analyzed. All quotes have been anonymized and participants are only indicated by the initial of the support group they belong to (eg, “B” for Balakasi and “S” for Somba) and a sequential number (eg, B4) to prevent the identification of individuals, in particular the few male participants.

### Daily Applicability of Messages’ Advice

There was an overall agreement that the messages were well-received and that the advice they provided was being put into practice in daily activities. For example, caregivers reported that the messages prompted them to be “More hygienic and cleaner” (S11) and “Wash hands more frequently” (B11) to contain the spread of COVID-19, or to “Take my medications [ARV] daily” (N11), “Start taking dosage as recommended” (L15), or “Practice safe sex by using condoms” (S26) to decrease the spread and impact of HIV/AIDS. Most participants confirmed following the health advice in their day-to-day lives, with a few exceptions. Interestingly, the message that was least followed was the one relating to HIV/AIDS and emotional and social support, which was distributed in January 2021 (message ID3; Table 2). Specifically, although our participants admitted the importance of this aspect, only a minority indicated that they somehow applied the guidance received. The message contained information on how to have open conversations about HIV/AIDS with family members and other people living in the community; it highlighted how HIV/AIDS is a life-changing condition, but individuals affected by it should not be treated differently from healthy individuals. It also suggested how informal caregivers should turn to others for any questions, concerns, or anxieties they may have to give the person in their care the best possible support. The caregivers disclosed that discussing one’s emotional state or entering intimate conversations about HIV/AIDS with others, even family members, was either not considered a priority or was still perceived as not socially acceptable. On the other hand, messages that offered more practical guidance were eagerly followed, as confirmed by caregivers during the interviews. In fact, of the remaining messages, the ones most adopted in everyday life were those relating to HIV/AIDS and COVID-19 (message ID1, adopted by 75/94, 80% of the participants), followed by those on HIV/AIDS and nutrition (message ID2, 70/94, 75% of the participants), the message on first aid and safety in caring for patients with HIV/AIDS (message ID4, 47/94, 50% of the participants), and finally the one on ARV dosage, side effects, and myths (message ID5, 38/94, 41% of the participants). As noted earlier, we did not evaluate the uptake of the message on safe motherhood and HIV/AIDS (message ID6) through the individual interviews; however, it was discussed at the final summative workshop. Specifically, the participating informal caregivers reported a very positive response from their communities with respect to this message and that it proved to be particularly relevant because many women were pregnant at the time of the study.

We consider that the variations in the immediate uptake of the advisory messages were not because of attrition, as this did not apply to our study’s research design. Instead, interviewees stated that the variations reflect and relate to the applicability and feasibility of the advisory messages in terms of changes in the communities’ everyday habits. Specifically, at the time of the study, the COVID-19 pandemic was starting to have an impact in Malawi in terms of the number of cases and containment measures. As such, the timing and the clear and simple instructions included in the advisory message allowed informal caregivers to easily adopt the said advice as part of their daily routines. Similarly, the message on nutrition involved guidance on how to prepare and preserve food and how to include all food groups in diets, which entailed achievable and simple changes to long-established habits. On the other hand, the messages on first aid and ARV therapy entailed either the occurrence of an “incident” for the first one or lapses in medication adherence for the other, both of which imply a more “ad hoc” adoption. Comments in this regard included “I will tell others to take medication and protect themselves for a healthy good life” (C6) or “Tell others on safety for healthy and good life” (S12). However, it is worth noting that despite the reduced applicability of some of the messages in terms of immediate changes in everyday life, all 94 participants confirmed that all the messages were highly valuable in relation to their content. Indeed, all participants, both throughout the interviews and at the summative workshop, reflected that they had noted positive changes (described as either “good” or “very good”) in daily community life.

### Long-term Perceived Benefits of Messages

When asked about the relevance of the advisory messages in terms of their potential to improve quality of life over time, caregivers unanimously envisaged that these could have a considerable impact on their lives, as they had already made a marked difference in the short space of the study. Table 4 summarizes the most relevant improvements in everyday life routines experienced by the informal caregivers as a result of the health advisory messages. Our study was conducted during the first wave of the COVID-19 pandemic, therefore the messages relating to hygiene, mask wearing, and handwashing were particularly well-received and most widely applied, not only for the duration of the project but also throughout the rest of the pandemic (Kalua, personal communication, May 2022).

**Table 4:** Most important and relevant improvements that the advisory messages prompted in caregivers’ lives.^a^

Improved aspect	Frequency of comments (number of individuals)
Understanding of COVID-19 and the importance of following rules (eg, washing hands, wearing masks)	20
Better understanding of HIV/AIDS, its transmission, and ways to keep safe	19
Better understanding of nutrition principles and food preparation	16
Better understanding of ARV^b^ therapy	8
Better understanding of overall principles of healthy living	6
No aspect specified, as all messages are equally relevant	32

^a^The overall number of caregivers was >94, as several participants reported >1 improvement.

^b^ARV: antiretroviral.

As explained earlier, the pandemic was a new event in the lives of caregivers at the time of the research, and most participants had not yet reached a clear understanding of its consequences on their community and individuals. However, advisory messages proved useful in clarifying the most common strategies to contain the spread of the virus. Indicatively, participants mentioned “Washing hands and wearing masks can prevent Covid 19” (N1), “We are cleaner now as we wash hands frequently” (S22), and “I have learned good habits from such message” (S18). As all messages were related to HIV/AIDS and its management, participants found that they helped them understand the ways in which people can protect themselves from contracting HIV/AIDS, managing its course, and being safe. The general view was that, “thanks to the messages, more people will know about the disease and not get infected” (B6). Caregivers felt that the messages would also help people become more upfront about HIV/AIDS despite the strong social stigma still associated with it. In fact, comments such as “More people will open up about the disease” (L11) and “More people can talk more openly and start the [ARV] treatment” (C8) were common among the caregivers. However, with respect to perceived stigma, the message relating to emotional and social support was not widely debated, as discussed earlier. However, our participants commented on the positive impact that talking about the condition can have in the long term because it would encourage “More people to come in the open” (S24) and that “More people will open up about having the virus” (L14), which suggests the perceived potential for the message to encourage people to disclose their condition and initiate ARV treatment.

The messages on medication dosage and nutrition were also highly valued by caregivers, and the advice contained in such messages was perceived as feasible and timely. In particular, there is information on how to maintain a nutritious diet by integrating the use of local staple foods (eg, cassava, sweet potatoes, and green bananas) with fruits and vegetables to produce healthy and diverse dishes. Participants reported that such advice would help them to “...differentiate from the past and make good changes from now on” (B9).

However, most encouraging was that some participants reported having developed a new attitude toward everyday life situations and tasks because of advisory health messaging, as they fully appreciated the messages’ deeper overall meaning, that is, to “Bring good health to all” (N5) and “Improve life for all” (C4). They considered that regularly following the advice would “...result in good decision making” (N10) and “...help people to learn good habits” (N4). Participants in the summative workshop also reported a sense of ownership with respect to the intervention because they were involved in it from its inception and wanted it to spread beyond the people attending the support groups and felt motivated to share the key content of the messages with their families and local community (“We can inform others on healthy living” [S17], “I will be able to help others” [L9], and “I will tell others how to live healthily” [B8]). In relation to the wider dissemination of the messages once they had been first distributed to the support groups, all participants confirmed that they had shared and discussed the content with their family members, and 76% (71/94) of them also had conversations within their local wider community. This sharing attitude aligns with the uMunthu philosophy, embedded in the Malawian culture and which values interconnectedness, inclusion, and interrelationships, as further explained in the *Discussion* section and as presented in the study by Zamani and Sbaffi [27].

### Quality and Clarity of the Messages

Regarding the way in which the messages were formulated, 67% (63/94) of participants stated that the messages were of ideal length, 17% (16/94) found them too long, and the remaining 16% (15/94) considered them too short. However, when asked about what changes they would like to see in further messages, 89% (84/94) of the participants were satisfied with the current format. Among the remaining 11% (10 caregivers), suggestions for improvement were about the need for the messages to include more information (5 comments), the messages needing to be delivered only in Chyao instead of including Chichewa as well (2 comments), as most of the people in the sample only spoke this local dialect, shared phones with more caregivers in the support groups for the messages to reach out to more people (2 comments), and included messages that target younger people affected by HIV/AIDS (1 comment). Two more participants suggested that posters and leaflets could be used to reinforce the influence of oral messages by functioning as reminders in people’s homes. Two participants stated that the language used was only partially clear and that some of the terms could have been simplified or explained better. However, the remaining 92 (98%) of the 94 caregivers reported that the language used was simple and effective. When asked whether they could understand the messages in relation to their content, all participants confirmed that they could. In addition, 66% (62/94) of the participants expressed the desire to receive more messages, ideally as a continuing intervention, and as part of their regular activities within the support groups. To achieve this, caregivers advocated the involvement of the Malawi government and Ministry of Health to implement this kind of support at the national level.

## Discussion

### Principal Findings

This paper reported on the implementation of a community-based intervention aimed at supporting informal caregivers of people with HIV/AIDS in rural Malawi in their daily caring activities via the dissemination of HIV/AIDS-related advisory messages. An important finding was that caregivers showed genuine willingness to develop their caring role. In this study, caregivers have not been just passive beneficiaries, welcoming support, but rather they were actively involved and heard and they were able and willing to contribute to the formulation, delivery, and dissemination of a community intervention aimed at supporting them in their caring role via practical and achievable health advice.

The challenge of participatory research designs in general and CBPR in particular lies in maintaining the original meaning of the term “participatory,” which is defined as “design initiatives aiming at the construction of socio-material assemblies for and with the participants in the projects” [58]. The relatively small scale of the intervention discussed in this paper has facilitated such participation, as confirmed by other similar small-scale studies [59,60]. However, despite the scale, the study, through the advisory messages, resulted in informal caregivers’ satisfaction and perceived improvements in everyday health outcomes. To some extent, we consider that the increased uptake of and the overall high level of satisfaction with the health advisory messages are because most of the participants, support group leaders, and trusted community chiefs have been championing the intervention among their communities and during support group meetings. Most importantly, we noted that consulting and engaging with our participants and the abovementioned stakeholders from the conceptualization phase of the study resulted in informal caregivers developing a sense of purpose and finding their voice, as the format of the intervention was coformulated with them, as well as the topics of the messages. In other words, they developed ownership over the intervention and felt deeply involved with the progress of the project and with the research team. This is demonstrated by the active role played by caregivers in supporting the formulation of the messages and relaying to the research team, and what aspects would be relevant to other caregivers and their communities, as also reported in the literature [42,43]. As existing research explains [43,61], the key elements fostering this shift from mere recipients to active participants and contributors are as follows: (1) the existence of strong champions, which in this research were represented by the support group leaders who were involved in the inception phase of the intervention, providing their vision of how it should have been implemented; (2) the involvement of the appropriate people, here in the form of community chiefs and local organizations well trusted and respected by community members; (3) trust building, achieved via regular and frequent interactions of the research team with the participants to share the status of the project; and (4) the active use of participatory engagement strategies, including caregivers not only being involved in the design of the intervention but also being consulted on the relevance and applicability of each message to the local context.

We consider such a participatory approach to be most beneficial when engaging with underserved groups in particular. Indeed, co-designing activities and tools with the participants is useful and desirable when it comes to the implementation of effective community initiatives, as also demonstrated in the literature [62]. However, on the other hand, there is a risk of bias in research findings, in the sense that they are positively skewed, because of an overly positive attitude of participants toward a project. This should be considered a potential limitation, especially for small-sized studies such as ours, where small deviations can skew the results. However, we noted that despite this potential source of bias, the level of acceptance of the messages, coupled with positive perceptions about their usefulness and applicability (RO1), was high. Other studies that focused on small, targeted health-related interventions in Malawi [63,64] exhibited similar findings, thus providing confidence in the validity of our results.

A second important finding of this study relates to the extent of the adoption of health advisory messages and their immediate effect on caregivers’ daily routines (RO2). Participants valued the advice received and initiated a process of integrating the advice from the messages with respect to simple aspects of everyday life, such as paying more attention to personal hygiene, varying their diet to include a wider range of food, and following with more care the posology of ARV medications. One aspect that participants concurred upon was that the COVID-19 pandemic was affecting their community life because of cases of infected people being on the rise at the start of the study. Similar concerns have been reported in other studies [65]. This change was worrisome as caregivers understood that the virus would have a particularly negative effect on their loved ones affected by HIV/AIDS. Research has reported that people living in low- and medium-income countries found it difficult to follow public health advice for limiting the spread of the virus, particularly the social distancing aspect, owing to overcrowded households and communities [66]. The implementation of simple actions such as washing hands and wearing masks, the importance of which was particularly stressed in the first of our advisory messages (coupled with the informal and easy-to-understand tone of the message), helped the caregivers feel more in control as they were able to take small tangible steps to manage an otherwise overwhelming situation. Research on COVID-19 and its impact on people living with HIV/AIDS showed that, despite the quick response from the African Union when the first cases emerged, the second wave of the disease has been more severe, with higher rates of infection and death in most countries in the region, including Malawi [67]. Under these circumstances, the message on COVID-19 supported caregivers in developing a better understanding of the seriousness of the situation and thus contributed to helping them adopt simple containment measures. The uptake of the subsequent messages followed a similar pattern, except for that related to HIV/AIDS and emotional and social support. Although other studies have reported on people’s perceived benefits of openly discussing HIV/AIDS [68], our findings reflect a reticence experienced by caregivers (many of whom are also affected by HIV/AIDS) in sharing feelings and asking for emotional support owing to the stigmatization still associated with the condition.

Although earlier studies further confirm this [25,69,70], we note that our own findings are somewhat ambiguous. We posit that the limited engagement with the emotional dimension and sharing aspects of living with HIV/AIDS might be explained by the nature of our participants, that is, most women who are embedded in a culture that stigmatizes patients with HIV/AIDS, which results in gender inequality and makes women more susceptible to HIV/AIDS infection [6,71]. On the positive side, and with regard to the potential long-term benefits of the advisory message related to HIV/AIDS and emotional and social support, some caregivers noted that, in time, such messaging might encourage more people to disclose their condition, which would then lead to a larger uptake of ARV therapy and, ultimately, to a healthier society.

Overall, the long-term positive impact of the messages (RO3) was acknowledged by all caregivers who had already started a process of sharing knowledge and practices within both their families and the wider community throughout the study. As discussed in Zamani and Sbaffi [27], the Malawian society is driven by the uMunthu philosophy, which “advocates the moral value of the importance of collaboration in the face of crisis” [72]. According to the Malawian culture, whoever possesses the uMunthu attitude is capable of showing compassion and a community-centered sense of care for fellow human beings, which helps explain the caregivers’ willingness to share the messages with the wider community, as observed in this study. However, as a note of caution, although uMunthu is well rooted in Malawi’s way of life, people are still influenced by what is deemed as socially acceptable, which demands a re-examination of their relationships with others and their environment and which could also explain, for example, the still widespread reticence in disclosing and discussing the emotional burden associated with HIV/AIDS. In this participatory study, the distribution of health advisory messages only to members of existing caregiver support groups was considered logistically viable, but their wider dissemination relied heavily on the “I am because we are” philosophy, which reflects the strong inclusive nature of uMunthu. This approach contributed strongly to the success and impact of the project.

In addition, this paper offers further contributions to both knowledge and practice. First and foremost, it proposes an effective and sustainable form of support and practical health advice for our principal stakeholders, the informal caregivers of people with HIV/AIDS in a low-income country, in their everyday life, and role as caregivers.

Second, it contributes to the field of information studies by addressing the needs of marginalized and underserved communities in rural settings. A major strength of this study is that it addresses such needs by embedding the dissemination of health-related information in common social practices and using local resources as a conduit for such dissemination.

Third, it contributes to the ongoing, but still developing, discussion on participatory designs. Our community-based participatory design allowed for the full integration of the intervention with caregivers’ way of life and the local culture. Our approach required time and effort to build a long-lasting trusting relationship between us as researchers and the local communities. However, the benefits of such a process translated into (1) the keen uptake of the health advice by all participants, (2) the development of a sense of ownership with respect to the intervention, and (3) increased self-assessed health outcomes throughout the project and beyond. In other words, the community-driven approach of this study facilitated the design of an intervention that was desired and designed by the informal caregivers, and the formulation of advisory messages that were timely and relevant to their own circumstances.

### Limitations and Further Research

This study has several limitations. The first limitation derives from the limited size and timeframe of the intervention. Although the design of the project allowed for a considerable number of informal caregivers to take part in it, it would be advisable to extend the initiative to more diverse rural communities throughout the country to fully appreciate the differences in caring attitudes and needs. A longer program of information dissemination would also allow for the gathering of more robust indicators of the long-term impacts on the well-being of the communities. Another limitation is the limited scope of the health advisory messages, which in this study were restricted to HIV/AIDS-related topics. Future scaling-up of similar initiatives should consider including more lifestyle and general health advice, for example, in relation to physical activity, tobacco smoking, nutrition, and alcohol consumption. A third limitation derives from the limited amount of qualitative data gathered from the interviews; as discussed previously, in most cases the caregivers did not elaborate on their answers nor provided satisfactory insights on some specific choices they made. Future studies in this area should consider deploying a more in-depth data collection tool that would help shed light on the complex challenges experienced by caregivers. A fourth limitation is the low number of men participating in the research, which could have produced biased results and did not fully capture the perspectives of men carers. As much as this is considered a limitation, our sample was, however, representative of the composition of local support groups. Further research should be dedicated to the male carers’ perspectives and needs, with the aim of better understanding the issues associated with disclosure of HIV/AIDS status in relation to the effectiveness of similar interventions.

### Conclusions

In conclusion, this study reported encouraging results concerning the implementation of a short-term intervention to support informal caregivers of people living with HIV/AIDS in their caring role. The study, which involved the formulation and dissemination of 6 HIV/AIDS-related advisory health messages to informal caregiver support groups in rural Malawi, was designed with the full participation of the local communities. The main aim of the messages was to assist informal caregivers in their caring role and to facilitate a better quality of everyday life via simple and achievable advice. Their content revolved around HIV/AIDS and its management, and all participants reported, in varying degrees, a perceived improvement in their daily lives and fully understood the long-term benefits that following the health advice would bring to them, their loved ones, and the community. However, most considerably, the advice contained in these messages was not just fully adopted by the caregivers involved in the research, but it was further disseminated by word of mouth beyond the immediate members of the support groups, and thus, this intervention reached wider local communities, resulting in broader benefits.

Although considering the limitations that a small-scale study such as this study poses, its relevance is undeniable, as it demonstrates that health interventions formulated via the synergetic combination of a CBPR approach, a cost-effective solution to support individuals and their communities, and in full respect of local ethos and values, have the potential to bring positive health changes, particularly in resource-constrained settings where the central health care system struggles to provide integrated services to remote areas of the country. This research could be used as a gateway for the implementation of large-scale projects aimed at improving the welfare of marginalized communities and has the potential to be applied to contexts beyond health care.

## Appendix

Multimedia Appendix 1 Health advisory message 1: COVID-19 and HIV/AIDS.

Multimedia Appendix 2 Health advisory message 2: HIV/AIDS and nutrition.

Multimedia Appendix 3 Health advisory message 3: HIV/AIDS and emotional and social support.

Multimedia Appendix 4 Health advisory message 4: antiretroviral dosage, side effects, and myths.

Multimedia Appendix 5 Health advisory message 5: first aid and safety in caring for patients with HIV/AIDS.

Multimedia Appendix 6 Health advisory message 6: safe motherhood and HIV/AIDS.

Multimedia Appendix 7 Preinterview checklist and interview topic guide.

## Abbreviations

ARV: antiretroviral
CBPR: community-based participatory research
CHBC: community home–based care
NACC: Namwera AIDS Coordinating Committee
