# Supplementary material for: Promoting Well-being Among Informal Caregivers of People With HIV/AIDS in Rural Malawi: Community-Based Participatory Research Approach
Source: J Med Internet Res. 2023 May 11;25:e45440. doi: 10.2196/45440 (PMC10214120; doi:10.2196/45440)
Supplement: Multimedia Appendix 1 [file jmir_v25i1e45440_app1.pdf]

CORONA VIRUS DISEASE AND HIV/AIDS

THANK YOU, COMMUNITY CARE GIVERS OF RELATIVES WHO HAVE HIV/AIDS. WE KNOW THAT, APART FROM TAKING CARE OF SOMEONE WHO IS CRITICALY ILL, SOME OF YOU HERE ARE ALSO HIV POSITIVE. TODAY WE WILL DISCUSS ABOUT HIV AND CORONOA VIRUS, AND IN THE SUBSEQUENT WEEKS, WE WILL DISCUSS OTHER TOPICS ABOUT HIV. Today YOU ARE WITH ME DR YUSUF SAIDI, ONE OF THE DOCTORS FROM BICO IN MANGOCHI WHO WORK WITH PATIENTS WHO HAVE HIV/AIDS.

You can listen to this voice message on WhatsApp on your own, or as you meet in your caregiver's monthly meetings. Later we will be asking you about whether the messages were useful and in what way.

For today, let's start with Coronavirus.

WHAT IS CORONA VIRUS?

Is a disease caused by a virus and it affects primarily the respiratory organs (nose, throat, and lungs)?

HOW IS THE DISEASE TRANSMITTED?

Most of the time, it spreads when a sick person coughs or sneezes. This can spray droplets as far as 6 feet away. If you breathe them in or swallow them, the virus can get into the body. Some people who have the virus don't have symptoms, but they can still spread the virus.

You can also get the virus from touching a surface or object the virus is on, then touching your mouth, nose or possibly your eyes. Most viruses can live for several hours on a surface that they land. This new virus called Covid-19 can last for several hours or days depending on the types of surfaces. That's why it's important to disinfect surfaces to get rid of the virus and wash your hands for at least 20 seconds before and after bringing things into your home.

When the virus enters the body, it takes 1 to 14 days for someone to develop signs and symptoms.

WHAT ARE THE SIGNS AND SYMPTOMS OF THE DISEASE?

- Fever
- Cough
- Sneeze
- Stuffy/runny nose
- Shortness of breath
- Fatigue
- Body aches/pains
- Headache
- Sore throat
- Loss of taste/smell
- Nausea and diarrhea

WHAT ARE THE RISK FACTORS FOR SOMEONE TO GET CORONOA VIRUS?

Anyone can get Covid-19 and most infections are mild. The older you are, the higher your risk of severe illness. You also have a higher chance of serious illness if you have one of these health conditions:

- Chronic kidney disease
- A weakened immune system **(HIV)**
- Obesity

- Serious heart conditions such as heart failure
- Diabetes
- Asthma
- Liver disease

NOW WE ARE GOING TO TALK ABOUT HIV AND CORONA VIRUS AND HOW IT AFFECTS YOU AS A CAREGIVER TO SOMEONE WHO HAS HIV

CORONAVIRUS (COVID -19) AND HIV

People with HIV who are on effective HIV treatment have same risk for Covid-19 as people who do not have HIV, but an HIV patient might be at increased risk for severe illness. The risk for people with HIV getting very sick is greatest in:

- People with a low **CD4 cell count**
- People not at effective HIV treatment

So, it is advised /emphasized that it is very important to take your medications regularly and follow Infection prevention measures. This is the best way to keep your immune system heavy and prevent developing severe illness from Covid-19 and on the same time preventing the HIV infection from progressing to AIDS.

HOW DO YOU PROTECT YOUR SELF FROM CORONA VIRUS AND ALSO HOW TO PROTECT THE CLIENT THAT YOU TAKE CARE OF

- **Wash your hands often with soap and water or clean them with an alcohol-based sanitizer**. This kills viruses on your hands.
- **Practice social distancing**. Because you can have and spread the virus without knowing it. you should stay home as much as possible. if you do go out, stay at least 6 feet away from others.
- **Cover your nose and mouth in public**. Wear a mask to protect yourself or others from getting the virus. This is not a replacement for social distancing. You still need to keep 6-foot distance between yourself and those around you.
- **Don't touch your face**. Coronavirus can live on surfaces you touch for several hours. If they get on your hands and you touch your eyes, nose or mouth, they can get into your body.
- **Clean and disinfect**. You can clean first with soap and water but disinfect the surfaces you touch with chlorine often like tables and doorknobs.
- **Avoid handshakes**.

CORONAVIRUS TREATMENT

There's no specific treatment for Covid-19. People who get infected get supportive care (to ease their symptoms) depending on the severity of the disease from mild to life threatening condition. The good news is that a lot of people are recovering from Covid-19. As HIV/AIDS remains a high risk factor, ensure that you immediately report to a health worker every-time you or the person you take about has any symptoms of Coronavirus.

THIS MARKS THE END OF TODAYS TOPIC. IF YOU HAVE QUESTIONS OR YOU WANT CLARIFICATION, PLEASE SEND WHATSUP MEESAGE TO 0994080273
