# Supplementary material for: Promoting Well-being Among Informal Caregivers of People With HIV/AIDS in Rural Malawi: Community-Based Participatory Research Approach
Source: J Med Internet Res. 2023 May 11;25:e45440. doi: 10.2196/45440 (PMC10214120; doi:10.2196/45440)
Supplement: Multimedia Appendix 2 [file jmir_v25i1e45440_app2.pdf]

HIV/AIDS AND NUTRITION

Nutrition is generally defined as how any living organism changes and uses food for life.

Food is anything that a person eats or drinks. From food we get **nutrients** - the part of food that an organism must have for life and health. The saying that ''we are what we eat'' reflects the necessity of good nutrients for human health and survival.

IMPORTANCE OF GOOD NUTRITION IN PLWHIV/AIDS

HIV weakens the body's immune system. However, the food that we eat *contributes to production of antibodies hence maintaining the immune system to fight off infections*.

Essentially, *good nutrition slows the progress of HIV and facilitates effective response to treatment*. Thus it is important for PLWHIV/AIDS of all age groups to have a balanced diet.

AIDS may also cause significant weight loss. *Good nutrition also helps people with HIV maintain a healthy weight.*

FOODS GROUPS AND THEIR FUNCTIONS

ENSURE YOU HAVE THE RECOMMENDED 6 GROUPS OF FOOD IN YOUR DIET.

FOOD GROUP	FUNCTION
1. **Staple foods** include foods high in carbohydrates, like whole grain maize flour (mgaiwa), porridge, irish potatoes, cassava, sweet potato, rice, green bananas, millet, sorghum, yams, coco, and wheat.	Carbohydrates give us energy for living, working, playing, etc.
**2. Animal foods group** includes eggs, meat, milk products, fish, ngumbi, mbewa, etc.	The foods in this group contain protein and fat necessary for repairing the body and for growth. These foods also help muscles to stay strong and help the body to build new muscles. They work to build the walls of all structures in our body. Proteins are used to build hair, skin, muscles, etc.
3. **Vegetable group** includes dark leafy vegetables (mpiru, bonongwe, chisoso, therere, pumpkin leaves, kholowa, khwanya, chigwada, mushrooms, mkhwani, mkoka bwato(Mwamuna aligone), Denje , Limanda, etc.	These foods are rich in vitamins, minerals and water. They also contain fibre necessary for proper digestion. Vegetables are immune boosting foods. These are important for protecting our body from germs that are trying to cause disease or sickness in our bodies.
4. **Legumes group** includes ground nuts, soya beans, beans, peas, cowpeas, ground beans (nzama), and pigeon peas, mbelemende, etc.	These foods provide protein and carbohydrate. They provide energy for activities as well as protein for body building functions.
5. **Fruit group** includes oranges, lemons, and tangerines, bananas, pineapple, pawpaw, mangoes, masau, bwemba, malambe, masuku,	Fruits contain carbohydrates, vitamins and water. Fruits have an added benefit of fibre and other medicinal properties.

peaches, apples, guava, watermelon and many others.	
**6. Fats group** includes oil seeds (soybeans, groundnuts, and sunflower seed), avocado (pear), cooking oil, milk and milk products such as butter, margarine, yoghourt, meat, fish, and poultry.	In the body, fats are burned for energy and provide warmth for the body.

HEALTHY DIET FOR PEOPLE LIVING WITH HIV

It is important for people living with AIDS to maintain a good weight. This mainly depends on maintaining appropriate calorie (a measure of how much energy food or drink contains). The amount of energy as well depends on:

- Your age - e.g., growing children and teenagers may need more energy
- Your lifestyle - e.g., how active you are
- Your size - e.g., your height and weight can affect how quickly you use energy.

Generally, the recommended daily calorie intake is 2000 calories a day for women and 2500 calories for men

The basics of healthy diet are the same for everyone, including people with HIV.

- Food from the five groups: fruits, vegetables, grains, protein and dairy
- Eat the right amounts of the above-mentioned food groups to maintain a healthy weight.
- Foods low in saturated fat, salt and added sugar are strongly not recommended as these may cause other medical conditions such as diabetes, kidney problems and heart conditions such as high BPs.
- Some special cases like ***wasting syndrome*** in people living with AIDS may require high energy and fat diet than normally recommended hence they may get this from ready to use therapeutic food such as ***Chiponde.***

EFFECTS OF HIV/AIDS OR HIV MEDICINES ON NUTRITION

HIV and ARV's can sometimes cause nutrition-related problems. Some HIV-related infections such as oesophageal candidiasis may make it difficult to swallow causing malnutrition in return.
Side effects from medicines such as loss of appetite, nausea or diarrhoea may make it hard to stick to healthy diet and HIV regimen both contributing to poorer health.

FOOD SAFETY AND HIV/AIDS

Food safety is about how to select, handle, prepare and store food to prevent foodborne illness.
Because HIV damages the immune system, foodborne illness are likely to be more serious and last longer in people with HIV/AIDS than in people with healthy immune system.
For people with HIV/AIDS should at least follow these food safety ways to reduce risk of foodborne illnesses:

1. **Do not eat or drink the following foods:** raw eggs or food that contain raw eggs, raw or undercooked poultry, meat, and sea food and unpasteurized milk or dairy products and juices.
2. **Follow the four basic steps to food safety:**
    - **Clean** wash your hands, cooking utensils and cooking areas.
    - **Separate** this will help to prevent spreading of germs from one food to another. E.g. keep raw meat, poultry, seafood, and eggs from ready to eat food including fruits and vegetables.
    - **Cook** food should be cooked thoroughly.
    - **Chill** this is one of the methods of preserving food for those who can afford a refrigerator can freeze mostly meet products. For our setting people use local methods such as salting boiling and drying to preserve food.
