# Supplementary material for: Promoting Well-being Among Informal Caregivers of People With HIV/AIDS in Rural Malawi: Community-Based Participatory Research Approach
Source: J Med Internet Res. 2023 May 11;25:e45440. doi: 10.2196/45440 (PMC10214120; doi:10.2196/45440)
Supplement: Multimedia Appendix 3 [file jmir_v25i1e45440_app3.pdf]

HIV AIDS AND SOCIAL SUPPORT

SOCIAL SUPPORT FOR PERSONS LIVING WITH HIV /AIDS (PLWHA)

- HIV infection affects all dimensions of a person's life: physical, psychological, social and spiritual.
- HIV infection can often result in stigma and fear for those living with the infection, as well as for those caring for them and may affect the entire family.
- Social support can therefore help people and their carers cope more effectively with each stage of the infection and help PLWHA respond adequately to the stress of being infected.
- As a caregiver there are a couple things that you can do to help PLWHA.
- In the messages that follow we'll discuss some of the ways one can socially support this group of people.

1. TALK

- Have open, honest conversations about HIV.
- Follow the lead of the person living with HIV/AIDS.
- Take time and do not pressurize them, let them pick the topics and depth of the conversation about the infection.
- Being sensitive is key to making them understand that you'll do whatever it takes to help them regain their healthy or to living comfortably.
- It is important to have normal conversations as before. This will assure them that you see them as the same person as they were before.

2. LISTEN

- Living with HIV is life changing experience.
- Listen to the person you're taking care of and offer your support.
- Show them that you understand and share their feelings.
- Reassure them HIV is a manageable health condition. It's not the end of life has to go on.
- There are medicine that can treat HIV and help them stay healthy.

3. LEARN

- In order to offer adequate and meaningful support one has to familiarize themselves on what they are dealing with.
- In this case one has to know what HIV and/or AIDS is, transmission, preventive measures, treatment options and how to stay healthy, just to mention a few.

- It is ultimate important for a care giver to understand as well that the one suffering from HIV/AIDS may not want all the information right away.

4. ENCOURAGE TREATMENT

- As much as HIV treatment is strongly recommended for all living with HIV. However, some people may stop taking medications basing on several reasons, e.g., no significant changes, discouraged by other people and encouraged to take herbal medicine claiming they cure or may be just getting tired because of the pill burden and side effects.
- Encourage these people to see a healthy care provider so that they may either be re initiated on the same or different regimen basing on the clinical assessment, help them explain more about the regimens, how and when to take together with side effects that the drugs may bring about and treat any opportunistic infections that may arise.
- It is also important for a care giver to advise politely and sensitively that there is no cure for HIV/AIDS, only treatment that helps to suppress the reproduction of the virus and boost their immune system in return is available.

5. SUPPORT MEDICATION ADHERENCE

- It is important for people living HIV/AIDS to take medications every day and as prescribed.
- Care givers should be able to ask their clients/loved ones what they can do to support them (PLWHA) in establishing a medication routine and sticking to it.

6. GET SUPPORT

- As someone who is taking care of someone it is important to take care of yourself and get support if need be.
- Turn to others for any questions, concerns or anxieties you may have, so that the person in your care should focus on taking care of their own healthy.

WHAT IF A FRIEND TELLS YOU THEY HAVE HIV

- You might know someone who is HIV+. This person could be a family member, a friend or a co-worker.
- Here is how you could be supportive if one of the above was to disclose their status:

  I. **Acknowledge.** Thank them for trusting you with their private health information

  II. **Ask.** Some people are public with this information and others not. Appropriately ask if there is anything that you could do to help. One reason they may have chosen to disclose their status to you is that they need an ally or advocate, or they may need help with a particular issue or challenge. Ask whether other people know this information, and how private they are about their HIV status.

  III. **Reassure.** Let the person know, through your words or actions, that their HIV status does not change your relationship and that you'll keep this information private if they want you to

IV. **Learn.** Educate yourself about HIV. Don’t make assumptions and look to your friends for guidance.

FINALLY

- If you are the sexual partner of someone who living with HIV, you should get tested so that you know your own HIV status.
- If you test negative, talk to your healthcare provider about PrEP, taking ARV’s daily to prevent HIV infection.
- PrEP is recommended for people at high risk of HIV infection, including those who are in a long-term relationship with partner who has HIV.
- If you get Positive, get connected to HIV treatment and care as soon as possible.
