# Supplementary material for: Promoting Well-being Among Informal Caregivers of People With HIV/AIDS in Rural Malawi: Community-Based Participatory Research Approach
Source: J Med Internet Res. 2023 May 11;25:e45440. doi: 10.2196/45440 (PMC10214120; doi:10.2196/45440)
Supplement: Multimedia Appendix 4 [file jmir_v25i1e45440_app4.pdf]

WELCOME YOU ALL CAREGIVERS OF RELATIVES WHO HAVE HIV/AIDS, TODAY WE WILL DISCUSS ABOUT HIV MEDICATION, DOSAGE, ADVERSE EFFECTS, MANAGEMENT AND IMPORTANCE OF CONTINUING ARV. TODAY YOU ARE WITH ME DR YUSUF SAIDI, ONE OF THE DOCTORS WHO WORK WITH PATIENTS WHO HAVE HIV/AIDS.

Before we start discussing about our topic, let me first clarity some words that will frequently be mentioned throughout our discussion so that you must know their difference.

- **HIV** is the virus that gets into the body.
- **AIDS** is a group of serious illnesses and opportunistic infections that develop after the body is too weak to fight back.

WHAT ARE MYTHS, RUMORS, AND UNTRUTHS ABOUT HIV AND PERSON/PEOPLE LIVING WITH HIV?

- Traditional healers and holy water can cure people with HIV.
- A faithful couple in which both people are HIV-positive does not need to use condoms.
- Only promiscuous people get infected with HIV.
- Anyone with TB or pneumonia has HIV.
- An HIV positive woman must have been sleeping around.
- People living with HIV should never have sex again.
- Having sex with a virgin can cure AIDS when they are positive.
- Being diagnosed with HIV is a death sentence.
- You can tell if a person has AIDS by looking at them.
- Feeling better after starting ARV treatment means an HIV-positive person has been cured.
- Being HIV-positive means a woman should never have children.
- All children born to women with HIV will get infected with HIV.
- ARV is too strong for pregnant women to take, as it will hurt the baby.
- HIV infected infants will not live for very long.

WHAT DOES HIV DO TO THE BODY TO DEVELOP AIDS?

The immune system is the body's natural defence against diseases. The human body is made up of many tiny cells, Cells are the basic building blocks in our body – they give us energy and keep us healthy and alive. In a healthy person, the immune system fights off diseases that enter the body to keep the person healthy. A type of cell called the CD4 cell helps the body fight infections. Some people talk about the CD4 cells as "soldiers" that defend the body. HIV enters the blood stream and starts to attack CD4 cells. For a while, the CD4 "soldier" cells keep the HIV virus weak in the body but after some time the HIV becomes stronger than the CD4 soldier cells and keeps making more of the virus and attacking more of the CD4 cells. The HIV keeps reproducing and there is more and more of it in the body. This makes people more likely to get infections and makes it harder for the body to fight these infections because they don't have as many CD4 cells. Eventually, the HIV attacks so many of the CD4 soldiers, that there aren't enough to fight back. The body is attacked by infections and germs that the person can't fight off. These infections are what eventually make people develop AIDS.

WHAT ARE HIV RELATED CONDITIONS (INFECTIONS)?

HIV related conditions (infections) are the infections that make people living with HIV sick because the body's immune system is weakened and it cannot fight back. Remember as what have said above the impact of HIV on the CD4 "soldier" cells in the body – when the HIV attacks the CD4 cells,

the person has trouble fighting back when a virus or germ enters their body. people living with HIV, especially people not on ARV, can get many of HIV-related conditions. One of the best ways to live positively with HIV, whether a person is on ARV or not, is to prevent HIV related conditions in the first place and treat them right away if they do happen. Usually, a doctor or nurse will give patients medicines to prevent these infections. They will also give some medicines to babies born to mothers with HIV to help prevent the babies from getting sick. Eating nutritious foods frequently, drinking clean water, sleeping enough, and practicing good hygiene also help prevent infections.

The most common HIV-related conditions are:

- Tuberculosis (usually in the lungs; the person will have a bad cough, fever, and will lose weight; easily transmitted from person to person)
- Chronic fever
- Pneumonia or PCP (a very bad infection in the lungs that can develop quickly; causes coughing, weakness, shortness of breath; is often what causes death in a person with HIV if it's not treated)
- Meningitis (a deadly disease in the brain, can cause bad headaches)
- Some cancers like cervical, Kaposi's sarcoma and lymphomas
- Prolonged diarrhoea - Can cause dehydration and weight loss
- Chronic anaemia
- Skin problems (like rashes or shingles, warts, or sore lesions; can be caused from fungus and be very uncomfortable)
- Oral sores (very common among and can be very painful)
- Malnutrition and weight loss

NOW LET US DISCUSS ABOUT HIV TREATMENT (ARV)

As a caregiver it's also important to discuss with your patient some myths about ARV

HERE ARE SOME OF THE COMMON MYTHS OF ARV

- ARV is a last resort and is only good for people who are sick and dying.
- ARV can kill you on the inside even though it can make you gain weight and look healthy on the outside.
- Without ARV, there is nothing PLHIV can do to stay healthy.
- Pregnant women can't take ARV because it will hurt the baby.
- People can share ARVs with family members.
- Once you are feeling better on ARV, you can stop taking it.
- If you feel well enough on ARV, you can have sex without condoms because the HIV won't spread anymore.
- Traditional medicines are just as good as ARV.
- There are no ARVs for babies and children.
- There is nothing you can do about side effects of ARV.
- If the ARVs make you sick, you should stop taking them.

COMMON ARV REGIMENS

As a caregiver, it is important to talk with patients about which medicines the patient is taking, why the patient is taking the medicine, and how often and how the patient takes each one.

Common First Line ARV Regimens in Malawi: Though patients are usually on three drugs, they are often available together in one pill called a Fixed-Dose-Combination (FDC).

Key facts about ARV regimens:

- ARV requires combining **3 different medicines** that act differently in order to avoid development of drug-resistant HIV.
- ARV regimens are not changed unless there is a clear medical need. Unnecessary regimen changes limit future treatment options.

A. 1st LINE REGIMENS

These are the best. Patients can remain on the same 1st line regimen for many years if they are fully adhering.

All 1st line regimes:

- Are easy to prescribe and easy to take
- Have a low risk of side effects
- Require no lab monitoring for toxicity
- Patients with significant side effects the 1st line regimen are moved to an alternative regimen without delay

Examples of first line medications are:

I. 13A (TDF/3TC/DTG)
II. 5A (TDF/3TC/EFV)
III. 4A (AZT/3TC/EFV) OR 4P (AZT/3TC/EFV)

B. 2nd LINE REGIMENS

These are for patients who have confirmed treatment failure on **1st line regimen** (usually due to poor adherence in the past).

All 2nd line regimens:

- Contain a completely different class of ARVs
- Are more complicated to prescribe and to take
- Can have more side effects

Examples of second line medications are:

I. 7A(TDF/3TC/ATV/r)
II. 8A(AZT/3TC/ATV/r)
III. 9A/P(ABC/3TC/LPV/r)
IV. 10A(TDF/3TC/LPV/r)
V. 11A/P(AZT/3TC/LPV/r)

C. 3rd LINE REGIMENS

This is 'salvage therapy' and a last resort for patients failing on 2nd line in spite of good adherence.

All 3rd line regimes:

- Very expensive
- Can have more side effects and be difficult to take.

TAKING ARVS AND MISSED DOSES:

• ARVs should be taken after the same number of hours every day (e.g., every 12 or every 24 hours). Most ARV regimens can be taken in the morning and at night. It does not matter if they are taken before, after or with food.

• Missing a dose: what to do if a patient remembers to take his ARVs late?

- ✓ **Less than half-way** to the next scheduled dose: take the missed dose immediately and take the regular next dose at the normal time.
- ✓ **More than half-way** to the next scheduled dose: skip the missed dose and take the regular next dose at the normal time.

SIDE EFFECTS OF ARV

One of the hardest parts of starting ARV can be dealing with the side effects. A side effect is a reaction to the medicine in the body – it can be good or bad, expected or unexpected.

- ❖ Expert clients play an important role in helping patients to learn about, prepare for, and manage side effects.
- ❖ It's important for patients to know that starting ARV is not an immediate cure for feeling bad. The ARV will help them feel much better over time, but probably not right away.
- ❖ Most side effects will go away after a few weeks of starting ARV. During this time of initial side effects, the patient needs to take their medicine correctly, every single day.
- ❖ There is a difference between minor or mild side effects that will go away (and patient should keep taking the drugs) and more serious side effects that may mean the doctor or nurse will switch the drugs.
- ❖ **A patient should never make the decision alone to stop taking some or all of the medicines. This should only be done in consultation with health care workers at the ARV CLINIC**

SOME COMMON SIDE EFFECTS FROM ARV AND HOW TO MANAGE THEM

Nausea and vomiting: Usually goes away in 2-4 weeks. Expert clients should tell patients to:

- ❖ Take medicines with food.
- ❖ Eat small meals more often.
- ❖ Avoid fried, greasy and fatty foods.
- ❖ Avoid spicy foods.
- ❖ Drink a lot of clean, boiled water, weak tea, or lemon water.
- ❖ Don't drink much coffee or strong tea.
- ❖ See clinician if there is fever, vomiting, inability to drink, or stomach pains.

Diarrhoea: Usually will go away 2-4 weeks. Expert clients should tell patients to:

- ❖ Eat small meals during the day.
- ❖ Eat soft foods like rice and bananas.
- ❖ Avoid spicy, greasy, or fatty foods.
- ❖ Drink sips of clean, boiled water, weak tea, oral rehydration salts or lemon water.
- ❖ **See the doctor or nurse if there is blood or mucous in the diarrhoea, if there is fever, if diarrhoea occurs more than 4-5 times in a day or for 5 or more days in a row, or if the person loses weight.**

Headaches: Usually goes away in 2-4 weeks. Expert clients should tell patients to:

- ❖ Rest in a quiet, dark room.
- ❖ Put a cold cloth over the face and eyes.
- ❖ Avoid strong tea or coffee.
- ❖ **See the doctor or nurse if it doesn't go away with paracetamol; or if there is fever, vomiting, blurry vision, or convulsions**.

Rash and skin problems: Expert clients should tell patients to:

- ❖ Keep skin clean and dry.
- ❖ Only use mild soaps.
- ❖ Drink a lot of clean, boiled water to keep skin healthy.
- ❖ See the doctor or nurse if the itching is severe, the skin is peeling, looks infected (for example has pus), is blistering, or has open sores; also, if the patient has a fever or if the rash is in the eyes or mouth.
- ❖ If taking the drug Nevirapine, come to see the doctor if there is any kind of rash.

Can't sleep or has nightmares: Usually goes away 2-4 weeks (most common with patients taking Efavirenz). Expert clients should tell patients to:

- ❖ Take pills at bedtime.
- ❖ Avoid heavy meals before going to sleep.
- ❖ Avoid alcohol.
- ❖ Avoid foods or drinks with sugar or caffeine before going to sleep.
- ❖ Talk about feelings and worries with Expert Clients, friends, or family members.
- ❖ See the doctor or nurse if the person feels depressed or suicidal.

Tiredness: This can be caused by many things. Expert clients should tell patients to:

- ❖ Avoid alcohol and drugs.
- ❖ Do light physical activity, like taking a walk.
- ❖ Eat lots of fruits and vegetables and make sure to get enough iron.
- ❖ Take multivitamins.
- ❖ Try to get enough sleep at night and rest during the day if needed.
- ❖ See the doctor or nurse if they have an alcohol abuse problem or feel depressed.

Numbness or tingling feelings: Can be caused by taking D4T and DDI, but also other ARVs and TB medicines, or if there are other infections. Expert clients should tell patients to:

- ❖ Wear loose socks and shoes to protect the feet.
- ❖ Check the feet to make sure there are no infection or open sores.
- ❖ Keep feet uncovered when in bed.
- ❖ Soak feet in warm water and massage them if this feels good.
- ❖ Keep feet up.
- ❖ Don't walk too much at one time; take breaks.
- ❖ See the doctor or nurse if the condition persists or they can't walk, are in pain, or weak.
- ❖ Also, if they are on d4T, they can visit the clinic as the doctor may want to decrease the dose.

Yellowness of eyes and palms: See a doctor or nurse if a client presents this condition. Remember, patients should not stop taking their ARVs if they have side effects! Some side effects are common

and most will go away with time, but if the patient thinks that the side effects are serious, he/she should call or visit the clinic right away. The decision to stop or change ARVs should be made by the patient and a health care worker together. Expert clients should refer all patients/clients to the hospital whenever there is a problem or side effects which cannot be managed at home.
