# Supplementary material for: Promoting Well-being Among Informal Caregivers of People With HIV/AIDS in Rural Malawi: Community-Based Participatory Research Approach
Source: J Med Internet Res. 2023 May 11;25:e45440. doi: 10.2196/45440 (PMC10214120; doi:10.2196/45440)
Supplement: Multimedia Appendix 5 [file jmir_v25i1e45440_app5.pdf]

FIRST AID AND SAFETY WHEN TAKING CARE OF PERSONS LIVING WITH HIV/AIDS (PLWHIV/AIDS)

FIRST AID is the first and immediate assistance given to any person suffering from minor or serious illness or injury.

SAFETY is the condition of being protected from harm or other non-desirable outcomes.

In first aid situations, many people are worried about disease transmission. The risk of catching any transmissible diseases such as hepatitis B/C, HIV, etc., when giving first aid is far less than people think. And this risk is further reduced by taking precautions and applying basic hygienic measures.

It is critical for each of us and even more critical for first aiders to understand how HIV is and is not, transmitted.

WAYS OF HIV TRANSMISSION

- Blood and other blood products, other body fluids (semen, pre-seminal fluid, rectal fluids and vaginal fluids) and transplanted organs that contain HIV
- Contaminated needles or syringe (drug use and accidental needle stick)
- Exposure through broken skin
- From a mother infected with HIV to her child during pregnancy, childbirth, or breastfeeding (breastfeeding is a low risk, especially when taking an antiretroviral regimen)
- Unprotected sex with someone who has HIV (man to woman, man to man, and woman to woman)

It is your responsibility to protect yourself and others by taking preventive measures and reducing risks. You can do this by:

- Making sure that needles, syringes or other instruments are sterile if skin is to be pierced or cut
- Ensuring injection needles and syringes are never shared
- Making sure that safe blood, which has tested negative for HIV, is used for transfusion
- Choosing abstinence- no sex is safest
- Insisting on safer sex through the use of condoms

WAYS IN WHICH HIV IS NOT TRANSMITTED

- Giving first aid when good safety practices are followed
- Contact of blood and other body fluids on unbroken skin
- Giving blood if you are not HIV positive
- Caring for someone with HIV/AIDS when appropriate precautions are followed.
- Touching, shaking hands and hugging
- Mosquitos or any other biting insects
- Coughing and sneezing
- Food and water
- Air
- Toilet seats
- Sharing clothes and bedding
- Swimming with a person who has HIV/AIDS

UNIVERSAL PRECAUTIONS IN FIRST AID

- Wash your hands before and after providing basic first aid. If gloves are available, wash hands before and after use
- Wear PPE whenever possible
- Be attentive to avoid contact with blood, body fluids, or soiled items
- Be careful of broken glass or sharp objects near the injured/affected person
- Cover cuts or open skin with a clean, dry dressing. Chronic skin conditions may cause open sores. People with these conditions should avoid direct contact with any injured person who is bleeding or has an open wound
- Decontaminate surfaces that do not require sterilization between patients with hospital grade disinfectant solution

First saves lives. Give first aid to anyone in need without discrimination and treat them all with respect

1. GIVING MOUTH-TO-MOUTH VENTILATION TO/BY A PERSON LWHIV/AIDS

This lifesaving procedure should not be withheld through fear of contracting HIV or other infections. If the injured person is bleeding from the mouth or the first aider has open mouth-sores, direct contact with blood should be avoided.

- Use a clean cloth or handkerchief, if available, to wipe away any blood from the injured person's mouth and to ensure clear airways.
- Use simple face shields or pocket masks during mouth-to-mouth ventilation. If possible, first-aid kits should contain such shields or masks, or the first aiders should carry their own.

2. DEALING WITH PLWHIV/AIDS WHO IS BLEEDING

Bleeding can be life threatening. A person losing blood needs first aid to stop the bleeding.

- If possible, instruct the injured person that he/she can stop the bleeding by applying direct pressure to the wound himself/herself.
- If the injured person cannot stop the bleeding for any reason, you can use clean, thick cloth, clothing or any other suitable material as a barrier to stop the bleeding and to avoid direct contact with the blood. When this compressive bandage is not efficient or possible, apply proximal pressure on the main artery.
- If you have gloves with you, wear them, especially in case of mass casualties or fighting.

3. BEING IN CONTACT WITH HIV+ BLOOD

- If your hands are contaminated with blood from PLWHIV/AIDS, wash them thoroughly with soap as soon as possible.
- If another part of your body is splashed or contaminated by blood or body fluids, especially the eyes, wash or flush it with lots of water.
- If your skin is cut by any object that is contaminated with blood of PLWHIV/AIDS, wash the wound thoroughly with soap and water and apply a dry and clean dressing.

If you are worried that you have been in contact with any kind of infection, seek confidential medical advice, counselling and testing.

4. CLEANING UP HIV+ BLOOD SPILLS

- Spilt blood should be soaked up with absorbent materials such as a cloths, rags, paper towels or sawdust. These materials should be considered as contaminated waste and be put in plastic bags for disposal, or burnt or buried.
- The area contaminated with blood should then be washed with a disinfectant (household bleach – diluted 1:10 with water to give 0.1-0.5 per cent concentration) to clean the area. Wait for 10 to 15 minutes before rinsing the contaminated area.
- Wear general-purpose utility gloves, thick rubber household gloves or two pairs of ordinary gloves to avoid contact with blood when cleaning the contaminated area. Put the gloves in a plastic bag for disposal after use.
- If gloves are not available, use other suitable materials to avoid direct contact with the blood.

5. CLEANING MATERIALS CONTAMINATED WITH HIV+ BLOOD

Cloths or clothing that are contaminated with blood should be handled with great care.

- Wear general-purpose utility gloves or thick rubber household gloves if you have to handle contaminated cloths or clothing, which should then be disposed of properly, burnt or buried.
- If contaminated cloths or clothing need to be washed, use detergent and hot water (at least 70 degrees Celsius) and soak for at least 25 minutes, or use cooler water with a detergent suitable for cold-water washing.

Don't forget

- People who are HIV positive can remain healthy for a long time before they develop AIDS. But they can still infect others, so do not try to guess who might be infected.
- People living with HIV/AIDS have the right to receive first aid without discrimination.
- Concern about infection can work both ways – transmission from the injured person to the first aider or from the first aider to the injured person.
- Once outside the body HIV is fragile and does not survive for long.
- Unbroken skin is a good barrier to prevent direct contact with HIV. The virus cannot get through unbroken skin.

Protect yourself and tell others, including friends and family, how to protect themselves against HIV/AIDS.
