# Supplementary material for: Promoting Well-being Among Informal Caregivers of People With HIV/AIDS in Rural Malawi: Community-Based Participatory Research Approach
Source: J Med Internet Res. 2023 May 11;25:e45440. doi: 10.2196/45440 (PMC10214120; doi:10.2196/45440)
Supplement: Multimedia Appendix 6 [file jmir_v25i1e45440_app6.pdf]

SAFE MOTHERHOOD AND HIV

Safe motherhood means ensuring that all women have access to the information and services they need to go safely through pregnancy and childbirth.

COMPONETS OF SAFE MOTHERHOOD

- Family planning
- Antenatal care
- Obstetric care
- Postnatal care
- Abortion care
- STD/HIV control

SAFE MOTHERHOOD AND STD/HIV CONTROL

Almost all HIV infections among young children are passed to babies from their mothers during pregnancy, labour and delivery, or breastfeeding. If a woman becomes infected with HIV while she is pregnant or breastfeeding, she has a very high risk of passing the infection on to her baby soon after she is infected with HIV.

Mother-to-Child Transmission is the transmission of HIV from a woman living with HIV to her baby during pregnancy, labour and delivery, or during breastfeeding.

PREVENTION OF MOTHER-TO-CHILD TRANSMISSION OF HIV

Prevention of Mother-To-Child Transmission of HIV provide services to reduce the number of babies infected with HIV and to improve the health of women.

COMPONETS OF PREVENTION OF MOTHER-TO-CHILD TRANSMISSION OF HIV

- Prevention of HIV transmission from HIV positive mothers to their babies during pregnancy, labour and delivery, and infant feeding.
- Provision of treatment, care and support to women infected with HIV, their children, and their families.

PREVENTION OF MOTHER-TO-CHILD TRANSMISSION STRATEGIES DURING PREGNANCY

For the work of care giver, the core element is routine counselling. For women testing positive, the messages below are very important:

- Be sure to attend all scheduled antenatal visits – at least 4 during pregnancy.
- HIV-infected women, like all women, should deliver their babies in a health facility with trained midwives and health care workers. Trained midwives can monitor progress of labour and ensure any needed interventions or referrals are made for the health of the mother and/or baby as soon as possible. Because most HIV infections are due to mother-to-child transmission during labour and delivery, it is especially important that women with HIV deliver in a health facility.
- HIV-infected women, like all women, are also encouraged to attend the family planning service with their husbands (partners) as part of comprehensive health care.
- ARVs will be provided immediately for all pregnant women to help the baby stay HIV-free as well as to protect the woman's health.

- Inform the woman of any mother's groups, support groups or other social support in the community.

PREVENTION OF MOTHER-TO-CHILD TRANSMISSION STRATEGIES DURING LABOR AND DELIVERY

Most Mother-to-Child Transmission of HIV happens around the time of labour and delivery. Care givers need to work with the multidisciplinary team, and providers in the labour and delivery unit, to make sure women understand and access safe delivery care.

Care givers should help women to understand and access these services:

- Plan to deliver the baby in a health facility with a trained provider. Talk to partners and other family members about how important it is for the mother and baby to have a safe delivery.
- Encourage mothers with unknown HIV-status to get tested right away.
- Encourage mothers who did not start taking ARVs during pregnancy to take them during labour and delivery, according to the health care worker's instructions.
- Promote good infection prevention practices for all births – keep the delivery area clean, washing hands, cleaning instruments, etc.

PREVENTION OF MOTHER-TO-CHILD TRANSMISSION STRATEGIES DURING INFANT FEEDING

HIV is present in breast milk and can be transmitted to babies through breastfeeding. Most of the HIV transmission during breastfeeding happens when the mother or other caregivers practice mixed feeding for their babies. Mixed feeding means giving babies breast milk together with other liquids, like water, herbal mixtures, or juice or other foods like cow's milk, formula milk, or soft porridge. Exclusive breastfeeding means only giving the baby breast milk (Except for medicines prescribed by a doctor) up to 6 months. Care givers should emphasize that there is much lower transmission from HIV positive mothers to babies through breastfeeding where:

- The mother is on ARV and adheres well to treatment.
- The baby ONLY gets breast milk up to 6 months (exclusive breastfeeding).
- Breast infections are prevented and treated right away.
- Thrush (white spots, yeast) in the baby's mouth is treated right away.

Here are key messages about infant feeding for care givers to explain to mothers:

- HIV positive mothers should take ARV to reduce HIV transmission. Remember to take ARVs every day, at the same time to make them work best.
- Continue taking ARV throughout pregnancy, breastfeeding and after to protect the baby from HIV and keep the mother healthy.
- Breastfeed exclusively for the first 6 months of the baby's life.
- Introduce nutritious complementary foods once the baby reaches 6 months and continue breastfeeding until the baby reaches 2 years of life.
- Feeding recommendations are the same for all infants, regardless of HIV exposure or HIV infection status:
    - Give only breast milk up to age 6 months (no water, gripe water, dawale, bara, thobwa, mzuwa, etc.)
    - Give only medicines prescribed by a health professional
    - Gradually start complementing breast feeding with suitable hygienically prepared foods from age 6 months (such as Likuni Phala, fruits, vegetables, beans, ground nuts and soya)

- Wean child around age 24 months
    - Wean gradually over a period of 1 month (no rapid cessation)
    - Replacement feeding (formula) is NOT recommended unless women are unable to breast feed
- If one breast has a problem, feed on the unaffected breast. Express breast milk of the affected breast and discard to prevent breast engorgement and maintain milk flow.
- Don’t feed baby from infected breast. Go to health facility for treatment.
- Ask the health worker to ensure correct positioning and attachment to the breast to prevent breast problems.
- Even if the baby is tested and found to be HIV-infected, the mother should keep breastfeeding for as long as possible.
- Women should watch out for breast infections (cracking, sore nipples, strange discharge from nipples, pain, etc.) and come to the clinic right away if this happens.
- Attend monthly mother - infant pair follow-up for clinical and nutritional care.

PREVENTION OF MOTHER-TO-CHILD TRANSMISSION STRATEGIES DURING THE POSTNATAL PERIOD

Care for HIV-exposed infants

All babies born to women with HIV are exposed to HIV because they shared blood and fluids with the mother. This does not mean that all babies will become HIV- infected. In fact, many babies will escape HIV infection, especially if the mother and baby get care and treatment. It’s important to explain follow-up care to mothers and families very well and make sure that they are not lost after the baby is delivered. There is a lot that can be done to keep the baby healthy when it has been exposed to HIV and there are tests that can be done to tell if the baby itself is HIV-infected.

Here is a summary of the key steps in caring for HIV-exposed infants:

➢ Health workers will actively screen all children under 24 months for HIV exposure by enrolling all children born to and/or breastfeeding from HIV infected mothers as soon as possible. Health workers will do HIV test as soon as possible from age 6 weeks to detect perinatal HIV infection and to allow for early ART initiation. The visit schedule for HIV exposed babies should be as follows:

- Monthly visits until age 6 months. Visits should follow the immunization schedule so that mothers and infants do not have to come to the clinic too often.
- Monthly visits from age 6 to at least 24 months.
- The health worker will schedule more frequent visits if the child is not doing well.

➢ Follow up for HIV exposed infants is provided for all infants with the 1st visit scheduled at 6 weeks. This 1st visit includes a check-up, immunizations and start of cotrimoxazole (CPT) for babies born to HIV infected mothers. If early infant diagnosis is available, blood will also be taken for an HIV test and sent to specialized laboratory.

➢ An HIV-exposed infant will continue CPT until the health worker is sure that the child is negative.

➢ At 12 and 24 months of age, provided the child has stopped breastfeeding, the child should have an HIV test.

➢ If the child continues to breastfeed after 24 months, CPT is continued for 3 months after breastfeeding has stopped, and the child is given an HIV test 6 weeks after cessation of breastfeeding.

➢ In both situations, if the HIV test result is positive, the child will continue life-long CPT. If ARV therapy is started, CPT will still continue.

➢ Babies born to HIV positive mothers will be discharged only after the baby tests negative for HIV.

➢ Babies confirmed to be infected with HIV are referred to HIV treatment services for further assessment and ongoing care.

<u>PREVENTION OF MOTHER-TO-CHILD TRANSMISSION STRATEGIES DURING THE POSTNATAL PERIOD FOR THE MOTHER AND FAMILY</u>

➢ If possible, link women with community health workers who can visit them regularly in their homes in the days/weeks after birth. Women who have heavy bleeding or signs of infection should come back to the hospital or clinic right away. Babies who have a fever, are crying or sleeping all the time, or are not eating well should also come back to the hospital/clinic right away.

➢ Make sure that women are enrolled in the HIV Care Clinic and treatment programs, have adherence support, and are on/continue taking ARV.

➢ Counsel the mother and family on follow-up of the baby, including Cotrimoxazole prophylaxis after the baby is 6 weeks old, importance of routine childcare visits for immunizations, etc., and HIV testing options for the baby and the timing and meaning of these tests.

➢ Encouraging the mother to tell health care workers that she has HIV and that the baby has been exposed. This will help ensure that she and the baby get the right care at every visit.

➢ Provide emotional support.

➢ Discuss family planning options.

➢ Linkages to support groups, nutrition for the mother, and income generating activities.

For the baby:

➢ Make sure the baby goes for check-ups and gets immunizations on schedule.

➢ Make sure the mother tells all health care workers that the baby has been exposed to HIV.

➢ Look for and treat oral thrush in the baby.

➢ Make sure the baby is getting good nutrition and growing well.

➢ NVP syrup is given to all babies born to HIV infected mothers:

  - NVP syrup shields the baby from HIV infection during the riskiest time
  - Health workers give NVP syrup to the baby 24-hourly for the first 6 weeks of life
  - All babies should take NVP syrup for the same duration regardless of the mother's ARV regimen and regardless of if the mother was taking ARVs at all

- Counsel the mother on early infant diagnosis and testing the baby. The baby may need 2 tests:

Once the baby is 6 weeks old, a test is conducted to see if the baby is HIV- infected. The test tries to find out which children Aare HIV-infected as early as possible so they can get the care and treatment they need. Remember, even if the baby is HIV-infected, the mother should continue breastfeeding. The test will have to be repeated later, about 6 weeks after the baby is weaned from breastfeeding.
