# Supplementary material for: Promoting Well-being Among Informal Caregivers of People With HIV/AIDS in Rural Malawi: Community-Based Participatory Research Approach
Source: J Med Internet Res. 2023 May 11;25:e45440. doi: 10.2196/45440 (PMC10214120; doi:10.2196/45440)
Supplement: Multimedia Appendix 7 [file jmir_v25i1e45440_app7.pdf]

Participants' Interview Topic Guide

Pre-interview Monthly Survey

1. **Name______________________________**

2. **Age_______________**

3. **Gender**
   - □Male
   - □Female

---

4. **How many people are living in your house?____________________**

5. **Marital status**
   - □Married
   - □Partner
   - □Divorced/separated
   - □Widowed
   - □Never married
   - □Prefer not to say

---

6. **Level of education (what is the furthest level of education that you have completed?)**
   - □No schooling completed
   - □Primary education
   - □Secondary education (e.g. GCSE/O-level)
   - □Post-Secondary education and higher (e.g. College, A-Levels, NVQ3 or below, or similar)
   - □Prefer not to say

7. **Can you read?**
   - □Yes
   - □No

8. **Can you write?**
   - □Yes
   - □No

9. **What is your first language?____________________________**

10. **Employment**

What do you do for a living?___________________________

11. **Do you own a mobile phone?**

☐Yes

☐No

12. **What type of mobile phone do you own?**

☐smartphone

☐non smartphone

13. **Is this phone your own or you share it with the household and other family members?**

☐shared with others

☐personal phone

14. **Who is the person with HIV you are caring for (i.e. what is the relation between you and the person with HIV)?___________________________**

15. **For how long have you cared for the person with HIV (years/months)?___________________**

16. **Do you have other caring responsibilities (e.g. elderly members of the family, children, etc.)?**

☐Yes

☐No

17. **Who else are you caring for?_____________________**

18. **On a scale from 1 (very poor) to 10 (excellent), what is your current quality of life?**

Very poor 1 2 3 4 5 6 7 8 9 10 **Excellent**

19. **On a scale from 1 (not at all) to 10 (completely), how confident do you feel in your caring role?**

Not at all 1 2 3 4 5 6 7 8 9 10 **Completely**

20. **Subjective health - how do you feel on a scale from 1 (very poor) to 10 (excellent)?**

Very poor 1 2 3 4 5 6 7 8 9 10 **Excellent**

Monthly Interview Questions

Questions about the monthly advisory messages

A. What are your impressions on the XX [*mention the relevant message when applicable*] advisory message?
  a. Did you know any of the information included?
  b. If so, what did you know? How did you learn about it?

B. What piece/s of advice did you apply to your daily life since receiving the message?
  a. Why this advice specifically? If you did not apply any, please explain why?
  b. Did you see a difference in your daily life since applying that advice [*specify which one*]? For the better or for the worst?

C. Was it difficult to apply the advice [*from a practical point of view*]?
  a. For example, too many people to social distance? Not enough water/soap/time to wash hands as often as required? [*provide examples suitable to the relevant advisory message when asking this question*]

D. Did you understand the benefits of the advice? If so, what do you think the benefits are? If not, why not?

E. What do you think about the relevance of the advice to your daily life? Do you think it will make a difference in the long term? Why/why not?

F. Were there aspects of the advice you found particularly useful AND useless? Can you explain further?

G. Did you discuss/share the advice with other members of your family after listening to it?

H. Did you discuss it with the rest of the community? What do you think are the general impressions of the community about its relevance?

Questions about the accessibility of the monthly advice

I. When did you first listen to the advice?

J. Was the message clear? Why/why not?

K. Did you understand the full message? Were there parts you could not understand/follow?

L. In your opinion, are voice WhatsApp messages the best way to distribute the messages? Are there other distribution ways [*e.g. poster, written WhatsApp messages, etc*.] you would have preferred and that you would like to see in the future?

M. Was the language used of a level accessible to everybody in the community?

N. Was the message too short/too long?

O. What changes would you like to see in the future [*to the delivery of messages that I have not mentioned*]?

P. Would you like to add anything else?
